# CCR5-Δ32 gene variant frequency in the Nigerian and Zimbabwean populations living in North Cyprus

**DOI:** 10.4314/ahs.v22i1.44

**Published:** 2022-03

**Authors:** Basil Chukwuebuka Ndikom, Mahmut Cerkez Ergoren, Murat Sayan, Gamze Mocan, Umut Fahrioğlu

**Affiliations:** 1 Near East University, Faculty of Medicine, Department of Medical Biology, Nicosia, 99138, Cyprus; 2 Near East University, Faculty of Medicine, Department of Medical Genetics, Nicosia, 99138, Cyprus; 3 Near East University, DESAM Research Institute, Nicosia, 99138, Cyprus; 4 Kocaeli University, Faculty of Medicine, PCR Unit, Kocaeli, Turkey; 5 Near East University, Faculty of Medicine, Department of Medical Pathology, Nicosia, 99138, Cyprus

**Keywords:** CCR5-Δ32, HIV, Nigerian, Zimbabwean, CCR5, North Cyprus

## Abstract

**Background:**

The cystine-cystine chemokine receptor 5 (CCR5) is the primary HIV co-receptor involved in the viral entry process into human cells. The 32 bp deletion variant within the CCR5 gene (CCR5-Δ32) plays a very important role in viral recognition and progression of AIDS.

**Objective:**

The current study was aimed at evaluating the CCR5-Δ32 gene variation frequency in Nigerian and Zimbabwean populations residing in Northern Cyprus.

**Methods:**

A total number of 211 subjects (103 Nigerians and 108 Zimbabweans) were analyzed. Nigerian population was further analyzed with respect to the three major ethnicities: Igbo, Hausa, and Yoruba. Polymerase Chain Reaction was used to determine the CCR5-Δ32 gene variant status.

**Results:**

All studied subjects from both sampling groups were homozygous for the CCR5 wild type gene (CCR5-wt), meaning neither heterozygous nor homozygous genotypes of CCR5-Δ32 gene variant were observed.

**Conclusion:**

This study observed the absence of CCR5-Δ32 deletion gene in the Nigeria and Zimbabwean populations living in Northern Cyprus. These populations lack the genetic advantage over HIV infection and may also show a rapid progression towards AIDS. Additionally, these populations could impact the local gene frequency as these two populations interact more and more.

## Introduction

Acquired immunodeficiency syndrome (AIDS), which remains one of the complex public health challenges in the world, is a complex infectious disease that weakens the host immune response. Human immunodeficiency virus (HIV), which is the virus that causes AIDS, can be transmitted from one person to another through semen, vaginal secretion, infected blood, and mucous membrane, pregnant woman to the baby during pregnancy, childbirth, or breast feeding. AIDS is classified as a progressive deterioration of the immune system of the infected person. Numerous studies reported both the host genetic factors and the viral genetics as the main important determinates of HIV-1 infection.^1^ There is a progressive depletion of the CD4+ T lymphocytes which is the major target of the virus. The continuous rise in the number of people living with HIV makes it a great health challenge in the world. According to United Nations joint program on HIV/AIDS, more than 37.9 million are infected with HIV across the world in 2019, while in the Nigerian and Zimbabwean populations, 1.9 million and 1.3 million people are infected, respectively.^2^

HIV-1 enters the immune cells such as the macrophages and T cell via the CD4 protein. The virus requires a primary CD4 receptor and a chemokine co-receptor 5 (CCR5) receptor to gain entry into the cell. Upon attachment of the virus to the target cell, there is a cellcell interaction that is mediated by the host cell's CD4 antigen and the 20-glycoprotein on the outer envelope of HIV. These T helper cells are the main target of HIV because they possess high number of CD4 molecules on their cell surface, and this causes them to have a higher binding affinity.^3^ Other cells such as Langerhans cells, macrophages, dendrite cells, monocytes, and microglia brain cells also possess CD4 on their cell surface. HIV can be grouped into two types; T -tropic or the x4 strains, which infect T cells only and the M-tropic or R5 strains, that infect both the macrophages and the T cells. CCR5 is required for the entry of the virus into their macrophages which cause a conformational change in the 41-glycoprotein leading to the fusion of the virus to the cell membrane. As the virus is taken into the cell, uncoiling of the particles exposes the viral genome.^4^,^5^ Moreover, CCR5 antagonist have been widely used in HIV treatments.^6^,^7^ Recent studies revealed that CCR5 cytokine receptor have been upregulated in COVID-19 patients which emerging clinical trial for COVID-19 treatment.^8^,^9^

CCR5-Δ32 is a mutate allele of the CCR5 gene with a 32 bp deletion which makes the cells lose their binding site on the cell surface. This mutation is commonly found in the European and Western Asian populations with higher frequencies recorded in Northern Europeans.^10^ Individuals that are homozygous carries of CCR5-Δ32 mutations are resistant to HIV infections because they do not express a functional CCR5 receptor used by the HIV-1 to enter CD4 containing cells. Individuals that are heterozygous are associated with lower viral loads and a slower progression to AIDS. Chemokine receptor gene CCR5 and its mutation (-Δ32) have become the object of intense interest with its roles in the entry of HIV-1 into the target cells. Dean et al.,^11^ examined the progress of HIV to AIDS in the US population with different levels of exposure to HIV ranging from intravenous drug users, persons with hemophilia, and homosexuals, they observed that the heterozygous individuals of CCR5-Δ32 had a two year delay in the progression to AIDS when compared with the homozygous wild type.^11^ This shows that heterozygous CCR5-Δ32 (CCR5-wt/ CCR5-Δ32) are not immune from HIV-1 infection but have a slow progression to AIDS. Recently, a paper detailing the CCR5-Δ32 allele frequency in 87 countries have been published. DKMS (Germany, Poland and UK) which collects samples from potential hematopoietic stem cell donors have implemented the genotyping routine to newly registered donors.^12^ New techniques are being developed to knockdown CCR5 expression by gene therapy with the help of zinc-finger nuclease (ZFN), CRISPR/Cas9 and transcription activator-like effectors nucleases (TALEN) systems.^13^ Yu et al. have created a double knockout system for both the CXCR4 and CCR5 genes in the circulating CD4+ cells using the CRISPR/Cas9 system which could potentially lead to more functional HIV prevention.^14^ There are 87 HIV positive cases in Northern Cyprus.^15^ Considering the Nigeria and Zimbabwe have the highest number of HIV cases among world populations increase number of African populations within Northern Cyprus urges different preventive medicine strategies. Therefore, this current study was aimed to determine the CCR5-Δ32 allele frequency within Nigerian and Zimbabwean populations residing in Northern Cyprus.

## Materials and methods

### Study population

This study consisted of 103 Nigerians and 108 Zimbabweans subjects. The Nigerian population was further subdivided into three major ethnic groups in Nigeria: Igbo, Hausa, and Yoruba. There were no other restrictions set up for the study population. An ethical approval for the study was obtained from the University Scientific Research Ethics Committee (XXX/2019/72-891). Informed consent was obtained from each participant.

### Genotyping

The region of the CCR5 gene containing the Δ32 deletion was amplified using the following flanking primers; 5′-CAAAAAGAAGGTCTTCATTACACC-3′and 5-CCTGTGCCTCTTCTTCTCATTTCG -3′. The expected fragments from the wt and the Δ32 allele were 189 and 157 bp, respectively. The polymerase chain reactions (PCR) reactions were 25µl and were prepared using the 2X PCR Master Mix by Thermo Scientific (K0171) with the final primer concentration at 20pmols for each primer. The PCR protocol was same as Angelis et al..^16^ A homozygous wt individual will only display the 189 bp band, a heterozygous individual will display both the 189 and the 157 bp band and a homozygous mutant individual will only display the 157 bp band.

### Statistical analysis

The Hardy-Weinberg equilibrium (HWE) was evaluated by the goodness-of-fit χ2 test to calculate genotype distributions and allele frequencies, where a p<0.05 was considered to indicate significant disequilibrium.

## Results

### Nigerian cohort

Total 103 Nigerian subjects whose are currently residing in North Cyprus have been studied. This studied group was consisted of 60 male (58%) and 43 female (42%) Nigerians. A total mean age was 25.7. There was no statistically significant difference (p= 0.292) between the mean age of male (25.2 ± 4.8) and the mean age of female (26.2 ± 4.8) (Table 1). Total 108 Zimbabwean subjects whose are currently residing in North Cyprus have been studied. This studied group was consisted of 56 male (52%) and 52 female (48%) Zimbabwean. A total mean age was 24.6. There was no any statistically difference (p= 0.291) between the mean age of male (25.4 ± 4.8) and the mean age of female (24.6 ± 4.8) (Table 1).

**Table 1:** The table illustrates a total mean age and the mean ages of male and females in Nigerian and Zimbabwean cohorts

**Nigerian cohort**		
**Age (mean)**	25.7	*p value*
**Male**	25.2 ± 4.8	p= 0.292
**Female**	26.2 ± 4.8	
**Zimbabwean cohort**		
**Age (mean)**	24.6	*p value*
**Male**	25.4 ± 4.8	p= 0.291
**Female**	24.6 ± 4.8	

### Nigerian population is made up of different ethnicities

Hausa, Igbo and Yoruba. First group was 26 (24.5%) Hausa subjects with 16 (15.5%) males and 10 (9%) females, second group was 45 (44.5%) Igbo subject with 27 (26%) males and 18 (18.5%) females and the last group was 32 (31%) Yoruba individuals consisted of 17 (16.5%) male and 15 (14.5%) female subject. Table 2 shows the distribution of individuals according to their ethnicities in Nigerian cohort.

**Table 2:** The table shows the distribution of studied Nigerian individuals according to their ethnicities

Ethnic group	Number of sample	Male, n (%)	Female, n (%)
Hausa	26	16 (15.5%)	10 (9%)
Igbo	45	27 (26%)	18 (18.5%)
Yoruba	32	17 (16.5%)	15 (14.5%)
Total	103	60 (58%)	43 (42%)

### Genotyping results

The subjects comprised of 103 Nigerian of which of 60 male (58%) and 43 females (42%). Genotype distributions and allele frequencies of the CCR5 gene Δ32 variant are shown in Table 3. The Zimbabwean total population of 108 was made up of 56 male (52%) and 52 females (48%). Genotype distributions and allele frequencies of the CCR5 gene Δ32 variant are shown in Table 3. Out of the 103 Nigerians and 108 Zimbabweans sample assessed, all the sample were homozygous for the CCR5 wild type gene (CCR5 -wt) (100%), while none (0%) was homozygous for the CCR5-Δ32 (mutant gene), also no heterozygous was observed. Hardy-Weinberg Equilibrium could not be used for the allele distribution analysis for both populations (p= 0.00), X2=0.00).

**Table 3:** Genotype distributions and allele frequencies of the *CCR5* gene Δ32 variant in the studied Nigerian and Zimbabwean cohorts

**Nigarian cohort**	**WT/WT**	**WT/Δ32**	**Δ32/Δ32**	**X2**	***p-value***
Observed	103	0	0	0	0
Expected	103	0	0		
WT allele frequency	1%				
Δ32 allele frequency	0%				
**Zimbabwean cohort**	**WT/WT**	**WT/Δ32**	**Δ32/Δ32**	**X2**	***p-value***
Observed	108	0	0	0	0
Expected	108	0	0		
WT allele frequency	1%				
Δ32 allele frequency	0%				

## Discussion

CCR5-Δ32 codes for a truncated and non-functional protein variant of chemokine receptor CCR5. This allele plays a very important role in HIV-1 infection and also shows complex involvement in different processes of the immune system. In this study, we determined the CCR5-WT and CCR5-Δ32 allele frequency in Nigeria and Zimbabwean living in North Cyprus. This study shows that all the Nigeria and Zimbabwean participants living in North Cyprus were CCR5-WT homozygous (100%) wild type. This concurred with the findings of Solloh et al.,^12^ when they studied CCR5-Δ32 allele frequency in potential hematopoietic stem cell donors registered with three DKMS donor center in Germany, Poland, and United Kingdom from 87 different countries including 160 Nigerians.^12^ Our results are in agreement with their finding giving 0.00% CCR5-Δ32 allele frequency. However, Zimbabwean population was not included in their global study, which made the Zimbabwean population a great choice in our study.

This result also is in agreement with the results of Ekere et al, in which they got (100%) homozygous CCR5 - WT from all their participant in a research conducted at the University of Calabar, Teaching Hospital in Nigeria and there was also no CCR5-Δ32 allele detected in the population.^17^ This shows that there is low/ absence of CCR5-Δ32 mutation in the Nigerian and Zimbabwean populations. Higher frequency of CCR5-Δ32 mutation has been reported in Northern Europe and a gradual decline south and eastward is observed, with no or rare occurrence in Africa, Asia, Americas and Oceania. Solloch et al.,^12^ showed that Northern Europe especially the Baltic region of Sweden, Estonia, Finland, Belarus and Lithuania have a high frequency of homozygous CCR5-Δ32. Cities and regions with higher frequency of homozygousCCR5-Δ32 mutation includes Moscow, northern coast of France, Volga Ural region of Russian and Ryazan.^12^

The allelic distribution of the CCR5 gene wild type in other populations were: 98.21% in Greek Cypriots, 75.56% in Russian, 91.22% in Jordanian, 87.5% in Turkish, 97.16% in Syrian, 100% in Yemen and 97.9% in Kuwait, 100% in Sudanese and 100% in Kenyan.^18^,^19^ We have also determined the genotypic distributions and allelic frequencies of the CCR5 gene variations in the Turkish Cypriot population. They observed approximately 3.0% of allelic frequency of the CCR5 -Δ32 variation within the Turkish Cypriot population with no observed homozygous individual of CCR5 -Δ32 allele^20^.

The relatively higher frequency seen for the CCR5 gene wild type allele in the African continent, suggests that the CCR5-Δ32 allele is a fairly recent mutation in terms of human evolution.^21^ In this study the Nigeria population was chosen since Nigeria is the most populated nation in Africa with an estimated population of 200 million people. Also, we wanted to complement the data by Solloch et al.,^12^ by analyzing the ethnic subgroups of the Nigerian population.12 In our study we have seen that the ethnicity in the Nigerian population has no relation to the distribution of the CCR5 -Δ32 mutation in the country.

The significant absence of the CC5R -Δ32 allele from the Nigerian and Zimbabwean populations in this study shows that they will be vulnerable to HIV-1 infection because they lack resistance to the infection and progression of AIDS will be accelerated. These populations are also important to North Cyprus as there are many students from these African countries living in North Cyprus. Mixing of these two populations will surely affect the allele frequency of the North Cyprus population. This should serve as a greater awareness to the society because the lack of genetic resistance can lead to the wild spread of HIV-1 in the population if exposed to the infection.
